# Intraocular pressure spikes after trabecular meshwork strip peeling during hemi-GATT

**DOI:** 10.1007/s00417-026-07182-8

**Published:** 2026-03-11

**Authors:** Mehmet Murat Uzel, Ali Mert Koçer, Bekir Eren Aksoy, Gözde Hondur

**Affiliations:** Ankara Ulucanlar Eye Research and Training Hospital, Health Science University, Ankara, 06250 Turkey

**Keywords:** Hemi-GATT, Trabecular meshwork peeling, IOP spike, Open-angle glaucoma

## Abstract

**Purpose:**

To evaluate the effect of trabecular meshwork strip peeling (TP) during hemi-gonioscopy-assisted transluminal trabeculotomy (hemi-GATT) on the frequency of early postoperative intraocular pressure (IOP) spikes and surgical success.

**Methods:**

This retrospective study included 42 eyes of patients with primary open-angle or pseudoexfoliative glaucoma who underwent hemi-GATT with TP (*n* = 14) or standard hemi-GATT without TP (*n* = 28) at a tertiary glaucoma center. Hemi-GATT was intentionally selected as a planned primary surgical strategy and was not performed as a secondary procedure following failed circumferential GATT. An IOP spike was defined as postoperative IOP > 30 mm Hg or an increase of ≥ 10 mm Hg from the previous visit. Surgical success was defined as qualified (IOP ≤ 21 mm Hg and ≥ 30% reduction from baseline with or without medication) or complete (without medication). The primary outcome was the incidence and predictors of early IOP spikes; secondary outcomes included surgical success rates and related risk factors.

**Results:**

An IOP spike occurred in 11/42 eyes (26.2%) at 7.1 ± 4.8 days postoperatively, lasting 6.7 ± 5.1 days; mean peak IOP during spikes was 26.2 ± 5.0 mm Hg. All patients with an IOP spike experienced a decrease in IOP to ≤ 21 mm Hg within the first postoperative month. Spike frequency was lower with hemi-GATT + TP than with standard hemi-GATT (1/14 [7.1%] vs. 10/28 [35.7%]; *p* = 0.048). On multivariable analysis, higher baseline IOP increased spike risk (odds ratio [OR], 1.48; 95% CI, 1.08–2.01; *P* = 0.013), whereas TP reduced it (OR, 0.01; 95% CI, 0.01–0.38; *P* = 0.014). The mean follow-up period was 7.71 ± 2.03 months (range: 6–13 months). Qualified and complete success did not differ (92.9% vs. 89.3%, *P* = 0.769; 42.9% vs. 35.7%, *p* = 0.714).

**Conclusion:**

TP during hemi-GATT significantly decreases early postoperative IOP spikes and enhances early IOP control, while maintaining surgical success.

## Abstract (fr)

**Key messages:**

***What is known*:**

- Postoperative spikes in intraocular pressure (IOP) frequently occur following gonioscopy-assisted transluminal trabeculotomy (GATT).

- Excisional removal of the trabecular meshwork, as performed in goniotomy techniques like the Kahook Dual Blade, is associated to a reduced incidence of intraocular pressure spikes when compared to standard GATT.

***What is new*:**

- Peeling of the trabecular meshwork strip during hemi-GATT significantly decreased the incidence of early postoperative intraocular pressure spikes in comparison to standard hemi-GATT.

- Higher baseline IOP was recognized as an independent risk factor for IOP spikes, whereas the removal of the trabecular meshwork (TM) strip demonstrated a protective effect.

- TM peeling achieved better early intraocular pressure control while maintaining mid-term surgical success, supporting its role as a simple and effective modification to enhance postoperative stability.

## Introduction

Glaucoma is a leading cause of irreversible blindness globally, causing a significant socioeconomic burden in terms of direct healthcare costs and lost productivity [1, 2]. When drug therapy fails to achieve target intraocular pressure (IOP), surgical intervention is essential. Traditional filtration techniques, such as trabeculectomy, can provide significant IOP reduction; however, complications, such as bleb-related scarring, hypotony, and infection, as well as the need for intensive postoperative care, pose significant disadvantages [3, 4]. These limitations and the finding that reducing IOP, the only modifiable factor in glaucoma, limits disease progression have increased the application of minimally invasive glaucoma surgery (MIGS) [5, 6].

Gonioscopy-assisted transluminal trabeculotomy (GATT), first described by Grover et al. in 2014, represents conjunctiva-sparing MIGS [7]. GATT improves trabeculo-canalicular outflow by ab interno circumferentially cutting the trabecular meshwork and the inner wall of Schlemm’s canal, while preserving the ocular surface anatomy for possible future filtration operations. Long-term surgical success rates of 70–90% have been documented in many glaucoma subtypes, including primary open-angle glaucoma (POAG), pseudoexfoliative glaucoma (PXG), uveitic glaucoma, steroid-induced glaucoma, juvenile glaucoma, and congenital glaucoma [8–13]. However, early postoperative IOP spikes are common and represent the second most frequent complication following hyphema [13–15]. IOP spikes not only threaten to increase optic nerve damage in already compromised eyes but also predict long-term surgical failure [13, 15]. In GATT, a circumferential incision is made in the trabecular meshwork (TM), and when performed over 180 degrees, it is referred to as hemi-GATT, while in excisional goniotomy or Kahook dual blade (KDB) goniotomy, a strip of the TM is surgically removed. Although there are few studies comparing these two surgical methods in the literature, it has been shown that early IOP spikes occur less frequently in KDB [16–19]. Complete removal of the TM may reduce the likelihood of flap reattachment or acute post-trabecular outflow obstruction. Although circumferential (360°) GATT is widely performed, hemi-GATT has been increasingly adopted as a primary surgical option due to its comparable intraocular pressure–lowering efficacy and a lower incidence of postoperative hyphema. For these reasons, hemi-GATT was deliberately selected as the surgical approach in the present study.

Despite the clinical importance of increases in IOP, the effect of TM strip removal on IOP spike formation in hemi-GATT has not been comprehensively evaluated. In this study, we investigated the effect of TM strip peeling (TP) on the frequency and severity of early postoperative IOP spikes by comparing conventional hemi-GATT with a modified technique involving excisional removal of the TM strip.

## Methods

This retrospective comparative study was conducted at a tertiary eye care center in Turkey. The study protocol was approved by the institutional ethics committee and adhered to the tenets of the Declaration of Helsinki.

### Patient selection

Medical records of adult patients diagnosed with POAG or PXG who underwent hemi-GATT with or without TP between January 2024 and March 2025 were reviewed. Surgical intervention was indicated in eyes with progressive glaucomatous damage or uncontrolled IOP despite maximum tolerated medical therapy, or in cases of medication intolerance or nonadherence. Exclusion criteria were: (1) previous intraocular surgery or trauma (except cataract surgery performed ≥ 6 months before enrollment); (2) secondary glaucomas other than PXG, e.g., pigmentary, angle-closure, or neovascular glaucoma; (3) unreliable visual field (VF) testing; (4) irregular follow-up visits; and (5) postoperative follow-up period of less than 3 months. Patient assignment to the trabecular meshwork (TM) peeling or non-peeling groups was nonrandom and determined intraoperatively rather than by predefined baseline characteristics. TM strip peeling was performed only in eyes in which adequate angle visualization could be achieved and the trabecular meshwork could be safely grasped. In some cases, increased adhesiveness of the trabecular meshwork, intraoperative bleeding, or suboptimal gonioscopic visualization limited the feasibility of TM peeling; these eyes were therefore included in the non-peeling group.

### Preoperative assessment and data collection

All patients underwent a comprehensive ophthalmic examination before surgery. This included best-corrected visual acuity (BCVA) using a Snellen chart, slit-lamp biomicroscopy, IOP measurement with Goldmann applanation tonometry, gonioscopy with a Goldmann three-mirror lens, fundus examination, 24 − 2 SITA Fast visual field testing using the Humphrey Field Analyzer (Carl Zeiss Meditec AG, Jena, Germany), and retinal nerve fiber layer (RNFL) analysis via spectral-domain optical coherence tomography (Heidelberg Engineering GmbH, Heidelberg, Germany). PXG diagnosis was based on the presence of pseudoexfoliative material on the lens capsule, pupillary margin, or angle structures. Patient demographic data and diagnoses, number of preoperative glaucoma medications, number of glaucoma medications at the last visit, details of the surgical procedure, intraoperative and postoperative complications, and the presence, onset time, and duration of IOP spikes were recorded. Glaucoma staging was conducted based on the MD value obtained from the visual field assessment [20]. 

### Surgical procedure

**Fig. 1:**
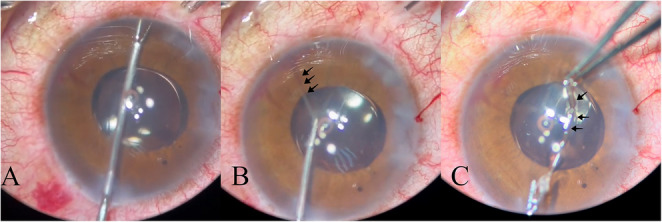
Intraoperative gonioscopic sequence of trabecular meshwork (TM) strip peeling during hemi–gonioscopy-assisted transluminal trabeculotomy (hemi-GATT). (**A**) Following completion of hemi-GATT, the elevated trabecular shelf is grasped with microforceps. (**B**) The TM strip is peeled within the anterior chamber using microforceps (black arrows indicate the TM strip). (**C**) The peeled TM strip is displayed on the corneal surface (black arrows)

All GATT surgeries were performed based on the surgical technique described previously [7]. Hemi-GATT was intentionally performed over 180° and was not related to failure of a previous 360° GATT. Surgeries were performed by the same experienced surgeon (M.M.U.) using a standard ab interno technique. After sterile preparation, with the surgeon sitting on the temporal side, two side ports were created in the superonasal or inferonasal quadrant using a 20-G microvitreoretinal blade. In right-eye surgeries, access was obtained through an inferonasal side port to perform superior hemi-GATT, whereas in left-eye surgeries, a superonasal side port was used to perform inferior hemi-GATT. This standardized approach was adopted to optimize surgeon ergonomics and gonioscopic visualization of the nasal angle. The anterior chamber was filled with a cohesive viscoelastic. A temporal corneal incision was created with a 2.8 mm knife. The microscope and the patient’s head were then tilted to allow adequate visualization of the nasal angle with a goniolens. A 5 − 0 Prolene suture, with the tip blunted by cautery, was inserted into the Schlemm canal via a nasal goniotomy under gonioscopic guidance. In the hemi-GATT group, the suture was pulled to complete a 180° trabeculotomy. In the hemi-GATT + TP group, the trabecular shelf tissue was excised with microsurgical forceps following trabeculotomy (Fig. 1). The study included eyes that had undergone at least one clock hour of TP. For patients requiring cataract extraction, clear corneal phacoemulsification and intraocular lens implantation were performed prior to hemi-GATT. The anterior chamber was irrigated to remove viscoelastic and refluxed blood, and viscoelastic was injected again to obtain a 25% anterior chamber fill.

### Postoperative management and follow-up

**Fig. 2:**
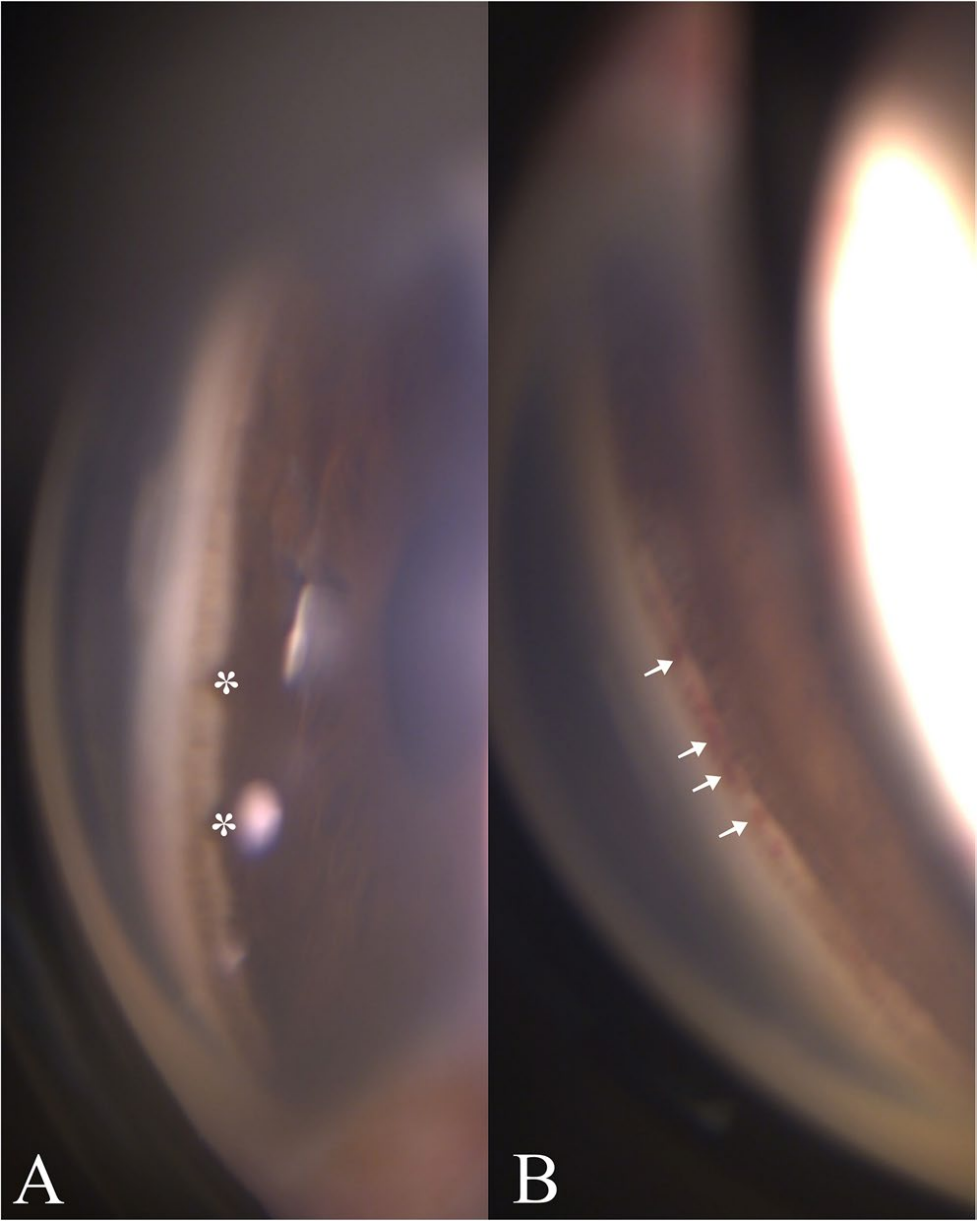
Postoperative gonioscopic appearance at postoperative month 6 following hemi–gonioscopy-assisted transluminal trabeculotomy (hemi-GATT) with and without trabecular meshwork (TM) strip peeling (TP). (**A**) Eye treated with standard hemi-GATT without TP showing residual trabecular tissue with areas of peripheral anterior synechiae (*asterisks*). (**B**) Eye treated with hemi-GATT with TP demonstrating a more clearly defined trabeculotomy cleft with visible blood reflux from collector channels (*arrows*)

All patients were prescribed topical antibiotics for 2 weeks, topical corticosteroids tapered over 4 weeks, and topical nonsteroidal anti-inflammatory drugs for 1 month. After surgery, anti-glaucoma medications (AGMs) were continued and then reduced or completely discontinued in the postoperative period according to the patient-specific IOP values. Postoperative evaluations were performed on day 1, day 3, week 1, week 2, month 1, month 3, and every 3 months thereafter. IOP was measured at each visit using Goldmann applanation tonometry. Patients developing IOP spikes (defined as an IOP > 30 mm Hg or a rise of ≥ 10 mm Hg compared to the previous visit) were monitored every 48 h until resolution. Treatment included topical AGMs, oral acetazolamide, and intravenous mannitol if needed. Postoperative gonioscopy was performed at the 6-month follow-up to assess the extent of the visible goniotomy cleft, which was recorded in clock hours (Fig. 2).

The primary outcome was early IOP spike. Secondary outcomes included surgical success by Kaplan-Meier analysis, defined as qualified success and complete success. The overall success of IOP reduction was defined as IOP ≤ 21 mm Hg and ≥ 30% reduction from baseline. Patients who met either success criterion without the use of topical IOP-lowering medications were designated as having complete success, while those who met the criteria with or without topical medications were considered to have qualified success.

### Statistical analysis

All statistical analyses were performed using SPSS version 23.0 (IBM Corp., Armonk, NY, USA). A two-tailed p-value of less than 0.05 was considered statistically significant. Categorical variables are presented as frequencies and percentages, and differences between groups were compared using the chi-square test or Fisher’s exact test as appropriate. Continuous variables were tested for normality using the Shapiro–Wilk test. Groups were analyzed using either the independent samples t-test or the Mann–Whitney U test, depending on the normality of their distribution. Kaplan–Meier survival analysis and the log-rank test were used to compare cumulative probabilities of surgical success between groups according to predefined success criteria. Patients lost to follow-up were censored in the survival analysis at their last visit. Risk factors associated with surgical failure were assessed using Cox proportional hazards regression. Differences in IOP and the number of AGMs between baseline and each postoperative visit were analyzed using linear mixed models with time as a repeated factor and Bonferroni correction applied for multiple comparisons. To evaluate factors associated with the development of early postoperative IOP spikes, univariate and multivariate logistic regression models were constructed for pre-specified baseline and postoperative variables. Variables with *p* < 0.20 in the univariate analysis were included in the multivariate analysis. Variables with *p* < 0.05 were retained in the final multivariate model.

## Results

A total of 42 patients who met the criteria were included in the study, with 28 in the hemi-GATT group and 14 in the hemi-GATT + TP group. The two groups were comparable in terms of age, gender, laterality, and glaucoma subtype (*p* > 0.05 for all). Baseline IOP was slightly higher in the hemi-GATT + TP group (32.21 ± 4.99 vs. 29.10 ± 5.69 mm Hg), but the difference was not statistically significant (*p* = 0.091). The baseline characteristics, including the number of AGMs, BCVA, visual field parameters, axial length, lens status, and rate of combined phacoemulsification, were comparable between the groups (*p* > 0.05 for all; Table 1). The mean follow-up period was 7.71 ± 2.03 months (range: 6–13 months). Twenty-two (52.4%) of the patients had moderate glaucoma and 20 (47.6%) had severe glaucoma.

**Table 1:** Baseline demographic and ocular characteristics of eyes undergoing hemi-GATT with and without trabecular meshwork strip peeling

	Hemi-GATT group (*n* = 28)	Hemi-GATT + TP group (*n* = 14)	*p* value
Age (years)	68.96 ± 9.01	68.28 ± 8.05	0.813
Gender (female) (n,%)	11 (39.3)	5 (35.7)	0.548
Laterality (right eye) (n,%)	14 (50)	4 (28.6)	0.161
Baseline IOP (mmHg)	29.10 ± 5.69	32.21 ± 4.99	0.091
AGM (n)	3.78 ± 0.41	3.50 ± 0.75	0.207
BCVA (logMar)	0.81 ± 0.49	0.50 ± 0.31	0.089
Cup to disc ratio	0.87 ± 0.14	0.86 ± 0.19	0.808
Mean deviation (dB)	−13.58 ± 5.45	−11.68 ± 4.85	0.260
Pattern deviation (dB)	7.47 ± 2.94	6.22 ± 2.16	0.169
Axial length (mm)	23.50 ± 0.67	23.95 ± 0.88	0.115
Lens status (phakic) (n,%)	20 (71.4)	8 (57.1)	0.279
Surgical procedure (phaco+hemi-GATT) (n,%)	19 (67.9)	7 (50)	0.215
Glaucoma type (PXG) (n,%)	18 (64.3)	11 (78.6)	0.282

*IOP* intraocular pressure, *AGM* anti-glaucomatous medication, *BCVA* best corrected visual acuity, *phaco+hemi-GATT* phacoemulsification+hemi gonioscopy assisted transluminal trabeculotomy, *PXG* pseudoexfoliatiın glaucoma, *TP* trabecular meshhwork strip peeling

An IOP spike was noted in 11 eyes (26.2%) on average postoperative day 7.09 ± 4.76 (range: 3–14). An IOP spike was observed in one patient (7.1%) in the hemi-GATT + TP group and in 10 patients (35.7%) in the hemi-GATT group (*p* = 0.048). All patients with an IOP spike experienced a decrease in IOP to ≤ 21 mm Hg during the first postoperative month. This was considered the end of the IOP increase, and the mean duration of the IOP increase was 6.72 ± 5.06 days (range: 2–21 days). The mean maximum IOP during the spike was 26.23 ± 4.98 mm Hg (range: 18–35 mm Hg).

Table 2 shows the factors influencing IOP spikes as determined by univariate and multivariate logistic regression analyses. Baseline IOP elevation (OR, 1.48; 95% CI: 1.08–2.01; *p* = 0.013) increases the risk of IOP spikes, while TM removal (OR, 0.01; 95% CI: 0.01–0.38; *p* = 0.014) reduces the risk of IOP spikes.

**Table 2:** Factors associated with early postoperative intraocular pressure spikes: univariate and multivariable logistic regression

Characteristics	Univarite analysis		Multivariate analysis	
	OR (95%CI)	*p* value	OR (95%CI)	*p* value
Age	1.04 (0.95–1.14)	0.350		
Gender	0.90 (0.21–3.26)	0.891		
TP	0.13 (0.01–1.22)	0.075	0.01 (0.01–0.38)	0.014
Baseline IOP	1.18 (1.01–1.37)	0.029	1.48 (1.08–2.01)	0.013
AGM	4.31 (0.54–34.18)	0.166	10.64 (0.42–269.03.42.03)	0.151
BCVA	2.96 (0.14–59.79)	0.479		
Cup to disc ratio	2.05 (0.02–183.81.02.81)	0.753		
Mean deviation	0.98 (0.86–1.12)	0.824		
Pattern deviation	1.00 (0.77–1.29)	0.985		
Axial length	1.12 (0.46–2.74)	0.789		
Lens status	1.20 (0.28–5.06)	0.804		
Surgical procedure	2.52 (0.61–10.27)	0.197	10.85 (0.97–121.24.97.24)	0.053
Glaucoma type	1.39 (0.32–5.97)	0.652		
Hyphema	0.57 (1.40–2.339.40.339	0.435		
Hemi-GATT superior/inferior)	1.90 (0.47–7.62)	0.365		

*TP* trabecular meshwork strip peeling, *IOP* intraocular pressure, *AGM* anti-glaucomatous medication, *BCVA* best corrected visual acuity, *hemi-GATT* hemi- gonioscopy assisted transluminal trabeculotomy

**Fig. 3:**
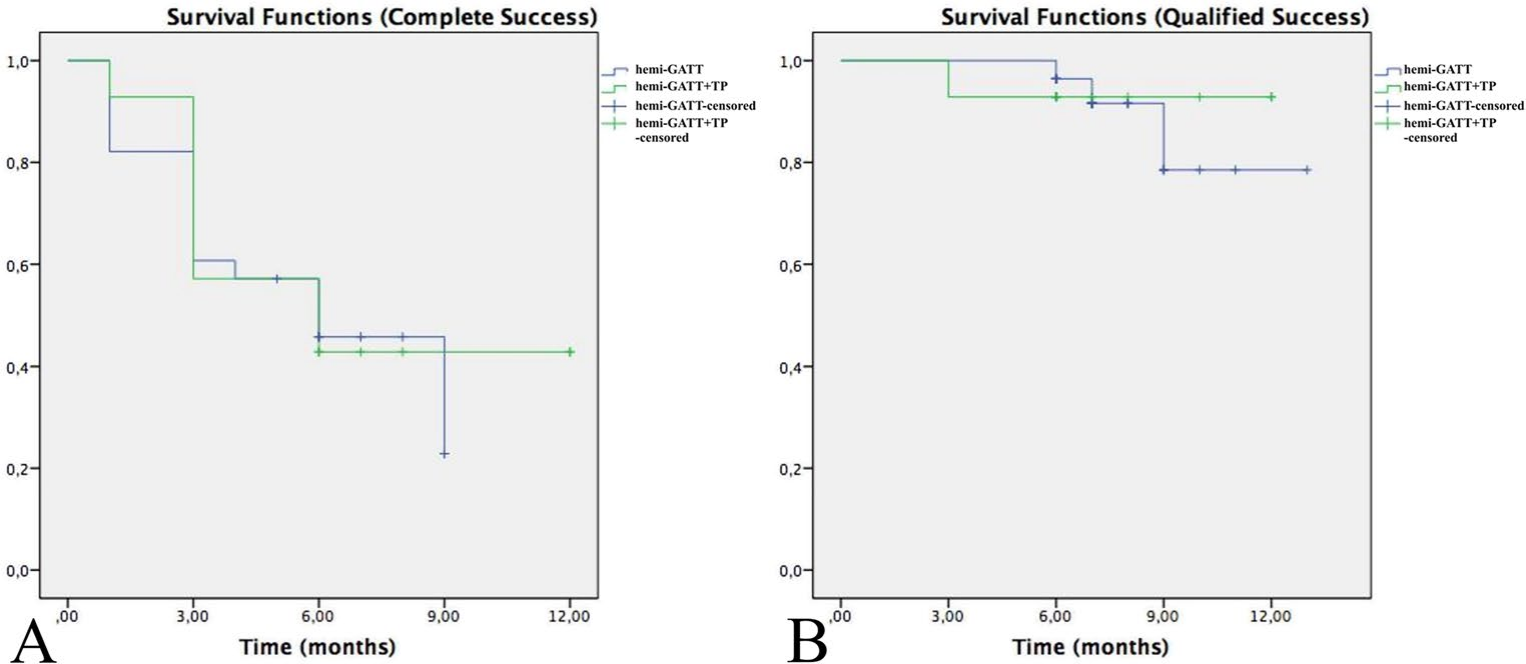
Kaplan–Meier analysis of surgical success after hemi–gonioscopy-assisted transluminal trabeculotomy (hemi-GATT) with trabecular meshwork strip peeling (GATT + TP) versus standard hemi-GATT. (**A**) Complete success: 12-month cumulative probability 42.9% with GATT + TP vs. 35.7% with standard hemi-GATT (log-rank *p* = 0.714). (**B**) Qualified success: 12-month cumulative probability 92.9% with GATT + TP vs. 89.3% with standard hemi-GATT (log-rank *P* = 0.769)

**Fig. 4:**
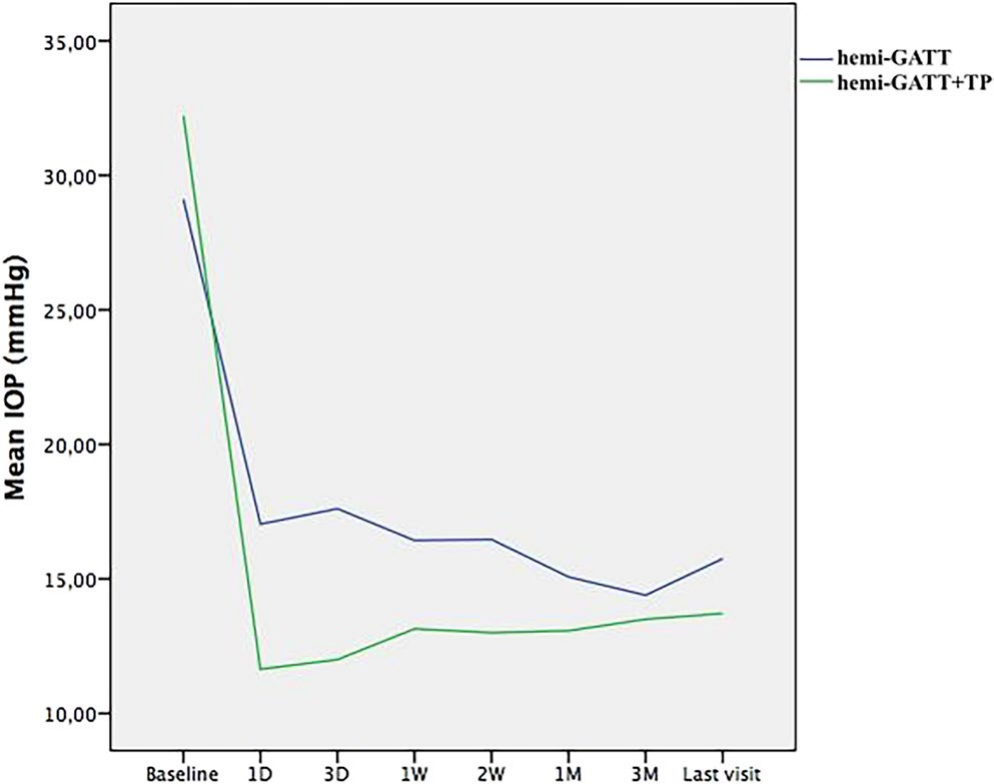
Longitudinal change in mean intraocular pressure (IOP) after hemi–gonioscopy-assisted transluminal trabeculotomy (hemi-GATT) with trabecular meshwork strip peeling (GATT + TP) versus standard hemi-GATT. D, day; W, week; M, month

In Fig. 3, Kaplan–Meier curves show the cumulative probability of complete and qualified success after surgery over a one-year period. The probability of qualified success was 92.9% in hemi-GATT + TP group vs. 89.3% in the hemi-GATT group (*p* = 0.769), and complete success was 42.9% in the GATT + TP group vs. 35.7% in the GATT group (*p* = 0.714). Mean IOP decreased from 29.10 ± 5.69 mm Hg preoperatively to 15.75 ± 5.94 mm Hg at 6 months postoperatively in the GATT group and from 32.21 ± 4.99 mm Hg preoperatively to 13.71 ± 3.77 mm Hg at 6 months postoperatively in the hemi-GATT + TP group. The mean IOP decrease between the groups was significantly different (*p* = 0.049). The mean percentage IOP reduction was 43.61% ± 25.28% in the hemi-GATT group and 56.62% ± 12.83% in the hemi-GATT + TP group (*p* = 0.033). The number of AGMs decreased from 3.78 ± 0.41 preoperatively to 0.71 ± 0.80 at 6 months postoperatively (*p* < 0.001) in the GATT group and from 3.50 ± 0.75 preoperatively to 0.71 ± 1.06 at 6 months postoperatively (*p* < 0.001) in the hemi-GATT + TP group; there was no significant difference between groups (*p* = 0.401). While no BCVA lower than baseline was observed in either group, an increase was observed in those who underwent cataract surgery. Postoperative IOP values were significantly lower in the hemi-GATT + TP group on day 1, day 3, week 1, week 2, and month 1 compared to the hemi-GATT group (*p* < 0.05, for all). The difference between the two groups was not significant after the first month. Figure 4 shows the change in IOP among the groups over time. Among all study variables, Cox stepwise regression survival analysis indicated that the presence of hyphema (hazard ratio, 2.120; 95% CI: 1.01–4.42; *p* = 0.045) increased the risk of surgical failure. Hyphema developed in 5 (35.7%) patients in the hemi-GATT + TP group and in 10 (35.7%) patients in the hemi-GATT group (*p* = 1.000). Two patients with surgical failure underwent trabeculectomy, one patient had Ahmed valve surgery, and one patient underwent micropulse transscleral cyclophotocoagulation with a diode laser. At 6 months, the visible goniotomy cleft extended for a mean of 3.85 ± 0.66 clock hours in the hemi-GATT + TP group and 3.42 ± 0.83 clock hours in the hemi-GATT group; the difference was not statistically significant (*p* = 0.103). In the GATT + TP group, the mean extent of peeling was 2.35 ± 0.84 clock hours. No significant correlation was observed between the extent of TM peeling and postoperative intraocular pressure at any follow-up time point (all *P* > 0.50).

## Discussion

In the present study, TP during hemi-GATT significantly reduced early postoperative IOP spikes compared with standard GATT. To our knowledge, this is the first study to investigate the effects of TP on IOP spikes in hemi-GATT.

The pathophysiology of IOP spikes is not clearly understood and is a fairly common complication of MIGS. Steroid use, the presence of postoperative hyphema, and advanced disease are the most commonly implicated factors [13–15, 21, 22]. IOP spike rates have been reported in the literature, ranging from 12% to 74% [13–15]. In our study, the IOP spike rate in the standard hemi-GATT group was 35.7% and was significantly lower in the hemi-GATT + TP group (7.1%). This wide range may be caused by the higher number of patients detected with intensive early postoperative follow-up in studies specifically evaluating IOP spikes and differences in IOP spike definition criteria. In our study, the IOP spike began on the 7th day on average and lasted for an average of 6.7 days, consistent with the literature [13, 15].

There are limited studies in the literature comparing GATT and excisional TM removal methods in terms of the presence of IOP spikes [16–19]. Rao et al., in their study evaluating microincisional trabeculectomy (MIT) and GATT with anterior segment OCT, found significantly fewer IOP spikes in the MIT group in which the TM was removed [17]. Furthermore, AS-OCT results revealed moderate-to-severe scarring in GATT eyes compared to MIT eyes, and a trench pattern was observed in more eyes as a healing response compared to the open gutter/saucer pattern in MIT eyes [17]. In a study comparing KDB and 360° GATT, no IOP spikes were observed after KDB goniotomy [16]. The authors interpreted this finding as being due to the removal of the TM strip, which created free space between the anterior and posterior TM leaflets. It should be acknowledged that trabecular meshwork strip peeling performed with microforceps is technically distinct from excisional goniotomy using dedicated devices such as the Kahook Dual Blade. While both approaches aim to remove trabecular meshwork tissue and reduce flap-related obstruction, differences in surgical precision, standardization, and the degree of tissue manipulation may influence local tissue trauma and healing responses. Therefore, comparisons with Kahook Dual Blade studies should be interpreted as conceptual rather than procedural, and direct equivalence between these techniques should not be assumed. Another report indicated that the presence of TM remnants correlates with increased AGM use and elevated postoperative IOP [23]. In our study, IOP was significantly lower in the first month in the hemi-GATT + TP group compared to the hemi-GATT group. Although IOP remained lower in subsequent months, the statistical significance was lost. These findings suggest that the presence of trabecular meshwork strip peeling, rather than the intraoperatively determined extent of tissue removal, may be the key factor associated with improved early postoperative IOP control, particularly within the first postoperative month. In contrast, the amount of TM peeling did not influence longer term IOP outcomes or surgical success. An IOP spike occurred in one patient who underwent TM peeling. TM peeling may increase proximal patency by removing the dysfunctional trabecular meshwork and potentially preventing the formation of a shelf-like flap, thereby possibly maintaining a clearer canal space and reducing the likelihood of flap inversion, reattachment, or early obstruction caused by shelf-centered fibrosis. However, the similar long-term results with the group without TP likely reflect the increased resistance in the distal pathway. TP may reduce the amount of TM remnants, or delay trabecular closure following healing processes, resulting in an additional decrease in IOP.

Only a few studies have examined the risk factors for IOP spikes [13–15, 21]. Ben Haim and colleagues reported that prolonged hyphema duration causes IOP spikes and that continuing the treatment regimen after GATT reduces the risk of IOP spikes and has a higher success rate [15]. In our study, we observed no difference in IOP spike development due to the presence of hyphema. We also gradually reduced AGMs in our postoperative treatment protocol. There are studies reporting that IOP spikes are more frequent in younger patients [15], but our study found no association with age. This difference may be due to the older age and similar age range of our patient group. It has also been reported that IOP spikes last longer in advanced-stage disease [13]. The study group comprised only moderate and advanced glaucoma patients, with no significant differences observed between the two stages. The present study found that preoperative higher IOP increases the risk of IOP spikes. It is plausible that the amount or rate of resistance in the distal and proximal trabecular pathways, particularly in glaucoma patients with elevated IOP, may play a role in this difference.

The rate of qualified success (92.9% in the hemi-GATT + TP group, 89.3% in the hemi-GATT group, *p* = 0.769) and the rate of complete success (42.9% in the hemi-GATT + TP group, 35.7% in the hemi-GATT group, *p* = 0.714) in the present study indicate that hemi-GATT is highly successful in open-angle glaucoma, similar to the literature [24–26]. In studies of hemi-GATT, IOP reductions vary from 37.9% to 59% over a one-year period. In our study, we observed IOP reductions of 43% in the hemi-GATT group and 56% in the hemi-GATT + TP group. These findings align with the existing literature indicating that the degree of trabeculotomy (hemi/segmental or circumferential) does not inherently affect long-term success. Furthermore, we noted a reduction in AGM usage, consistent with existing literature [24–26].

Current data suggest that IOP spikes, the presence of postoperative hyphema, disease stage, and first-month IOP levels are more important for success than the length of the goniotomy [24–26]. Studies comparing hemi-GATT and 360º GATT have reported higher rates of IOP spikes and hyphema with 360º GATT [24, 26]. Although many series do not demonstrate a simple presence/absence relationship between hyphema and IOP spikes, longer or more severe bleeding appears to be clinically significant. In our study, the presence of hyphema was also a significant factor in surgical failure. The presence and long duration of hyphema may accelerate the healing process at the goniotomy site or create obstruction distally, leading to surgical failure in the long term. Studies in the literature claim that both the presence and duration of an IOP spike are associated with an increased risk of surgical failure [13, 15]. The reason the presence or duration of an IOP spike was not a factor in our study may be explained by the early response to spike therapy and the fact that the patients had different glaucoma etiologies. Studies show that proximal trabecular pathway obstruction in PXG increases IOP [27], making GATT surgery more successful in PXG than in POAG [8, 9], supporting this conjecture. In our study, PXG patients represented the majority in both groups. Recent studies demonstrate the efficacy of 180º and 360º GATT in advanced disease [25, 28]. Our study cohort consisted primarily of moderate and advanced patients. Although evidence in the literature is limited, inferior hemi-GATT is preferred because of the higher number of collector channels in the lower hemisphere [29, 30]. In the present study, applying GATT in the upper or lower hemispheres did not affect surgical success, consistent with the literature [31].

This study has several strengths. To our knowledge, this is the first study to investigate the effect of TP on IOP spikes after hemi-GATT. It includes a uniform surgical technique performed by a single experienced surgeon, pre-specified IOP spike definitions with close early follow-up, and multivariate modeling of IOP spike predictors. There are several limitations of our study, including the study’s retrospective design, the group imbalance and modest sample size (28 vs. 14 eyes), and the short- to medium-term follow-up. This may limit statistical power and the precision of effect estimates. In particular, the low number of early IOP spike events in the TM peeling group may reduce the robustness of multivariable regression analyses, and the results should therefore be interpreted with caution. Accordingly, the present findings should be considered exploratory and hypothesis-generating rather than confirmatory. Nevertheless, as the first study to comprehensively evaluate the effect of trabecular meshwork strip peeling during hemi-GATT, the observed associations provide a relevant preliminary clinical signal that may help inform the design of future prospective studies. Angle assessment with AS-OCT was not performed preoperatively and postoperatively, so we were unable to visually document the effects of TP on angles. The patient groups had different glaucoma types; however, the analyses revealed that the glaucoma type had no effect on the IOP spike or surgical success. Although we included patients who underwent at least 1 h-quadrant TP, variability in TM tissue adhesion across patients challenged our ability to standardize the amount of TP. Similar to the lack of a significant effect on surgical success between 180° and 360° goniotomy, we believe the degree of TP has no significant impact on preventing IOP elevations. Because TM strip peeling could not be performed in all eyes due to intraoperative factors such as trabecular adhesiveness, bleeding, or limited angle visualization, group allocation was nonrandom, which may introduce selection bias.

In conclusion, TP during hemi-GATT leads to significantly lower early IOP spikes and better early IOP control. Medium-term surgical success was comparable to standard hemi-GATT. These findings support the mechanistic premise that removal of residual TM reduces flap-related obstruction and fibrotic closure during early healing, as demonstrated in the literature. Prospective randomized studies in standardized patient groups are needed to investigate the long-term efficacy of TP and its effects on the angle.

## Data Availability

No datasets were generated or analysed during the current study.
